# Clinicopathological Significance of AKT1 and PLK1 Expression in Oral Squamous Cell Carcinoma

**DOI:** 10.1155/2022/7300593

**Published:** 2022-06-17

**Authors:** Er-Can Sun, Shuang-Shuang Dong, Zhi-Jun Li, Chang-Xue Li

**Affiliations:** ^1^Department of Stomatology, Shihezi University School of Medicine & the First Affiliated Hospital to Shihezi University School of Medicine, Shihezi, 832002 Xinjiang, China; ^2^Department of Pathology, Northern Jiangsu People's Hospital Affiliated to Yangzhou University/Clinical Medical College, Yangzhou University, Yangzhou, Jiangsu 225000, China

## Abstract

**Purpose:**

Oral squamous cell carcinoma (OSCC) is the sixth leading cause of cancer-related death worldwide and is characterized by metastasis and recurrence. We aimed to evaluate the expression of AKT1 and PLK1 in OSCC and identify their correlation with the clinical and histological features and prognosis of patients with OSCC.

**Methods:**

Tissue samples were collected from 70 patients with OSCC and 50 patients with normal oral mucosa. The expression levels of AKT1 and PLK1 in OSCC tissues and normal oral mucosa were detected by immunohistochemistry. The chi-square test was used to identify correlations between the expression levels of AKT1 and PLK1 with patients' clinicopathologic characteristics. Survival analysis was assessed by the Kaplan–Meier method. Spearman's rank correlation test was used to determine the relationships between AKT1 and PLK1 expressions. The bioinformatics database GEPIA was used to verify the experimental results.

**Results:**

The chi-square test and Fisher's exact test showed that the positive expression rate of AKT1 and PLK1 in OSCC tissue was significantly higher than that in the normal oral mucosa (*P* < 0.05). PLK1 expression levels were significantly correlated with tumor stage and size (*P* < 0.05). Kaplan–Meier analysis showed that the survival time of AKT1 and PLK1 with high expression was significantly shorter than that of patients with low expression (*P* < 0.05). Spearman's rank correlation test showed a strong correlation between AKT1 and PLK1 expression in OSCC tissue (*R* = 0.53; *P* < 0.05). GEPIA bioinformatics database analysis results show that the expression and overall survival of AKT1 and PLK1 analysis and the correlation analysis of AKT1 and PLK1 were consistent with experimental results.

**Conclusion:**

AKT1 and PLK1 expressions are associated with the occurrence and progression of OSCC and may be used as diagnostic and prognostic indicators of OSCC. There may be a correlation between AKT1 and PLK1 in OSCC tissue.

## 1. Introduction

Oral squamous cell carcinoma (OSCC) is one of the most frequent neoplasms worldwide, showing very aggressive behavior, propensity for lymph-node metastasis, and a lousy prognosis [1, 2]. OSCC includes cancers of the tongue, lip, bottom of the mouth, gingival, buccal, posterior molars triangle, and hard palate [3]. Some dysplasia areas in the oral are high-risk factors for OSCC, such as leukoplakia, erythroplakia, erythroleukoplakia, oral lichen planus, oral submucous fibrosis, and oral dysplasia [4]. The prevalence of OSCC increases with age [3]. There are more than 200,000 new confirmed cases of OSCC in the world every year [5]. Despite improvements in surgical techniques and chemotherapy, the prognosis for OSCC remains poor, with a 5-year overall survival (OS) rate of only 64.4% [6]. Surgery combined with chemotherapy can improve OS in patients with OSCC, preoperative chemotherapy can shrink the tumor, and postoperative chemotherapy can help prevent tumor recurrence and metastasis. However, after surgery, radiotherapy, and chemotherapy, most patients will have severe toxic and side effects such as local defects, malformations, functional disorders, and drug resistance [7]. Targeted therapy emphasizes the treatment of diseases at the molecular level, with high targeting and specificity, which dramatically reduces host toxicity and improves the quality of life of patients [8]. In the past two decades, targeted therapy has become a new approach to treating various human diseases, including cancer [9]. Several targeted anticancer agents have been successfully introduced into clinical practice [10]. However, OSCC is a multifactorial, multistep, multigene genetic disease, and its molecular pathogenesis is still not fully understood. Therefore, it is of great clinical significance to further study the pathogenesis of OSCC and find practical molecular markers to predict the prognosis and for targeted therapy of OSCC.

Polo-like kinase 1 (PLK1), a member of the PLK family, is a serine/threonine protein kinase and is widely recognized as an oncogene. PLK1 plays a crucial role in the cell cycle and drives cell proliferation by promoting mitosis and cytokinesis [11–13]. In addition, PLK1 also has roles in meiosis, including regulating cancer cell invasiveness and preventing cancer cell apoptosis [14]. Recent studies have shown that PLK1 overexpression can promote the development of breast cancer, renal cell carcinoma, and gastric cancer [15–17]. AKT (also known as protein kinase B or PKB), with the subtypes AKT1, AKT2, and AKT3, is a critical intracellular kinase in the PI3K/AKT signaling pathway. It has significant roles in cell differentiation, growth, and targeted therapy of many human malignant tumors [18, 19]. The PI3K/AKT signaling pathway plays a major role in basic cell activities such as cell metabolism, cell growth, cell proliferation, apoptosis, and angiogenesis [20]. Once this pathway was discovered, many medical studies explored it [21].

However, only a few reports exist on the relationship between the PI3K/AKT signaling pathway and PLK1 in OSCC. In this study, the expression of AKT1 and PLK1 in OSCC tissues and normal oral mucosa was compared, and the relationship between AKT1 and PLK1 and OSCC clinicopathology and prognosis were discussed. Our study explores their potential value as biological and prognostic markers for the occurrence and progression of OSCC.

## 2. Material and Methods

### 2.1. Patients and Tissue Samples

A total of 106 tissue samples from patients with OSCC and 73 normal oral mucosa samples from patients treated in the First Affiliated Hospital of Medical College of Shihezi University in Xinjiang province from 2008 to 2012 were collected as the case group and the control group, respectively. Normal oral mucosa samples were taken from gingival, tongue, and buccal mucosa. All patients received no treatment before surgery and had no other medical history [22]. From the initial samples, 70 OSCC tissues with complete clinicopathological and follow-up data and 50 normal oral mucosa specimens were selected for the study. The research group conducted follow-up once a year, and the follow-up data of this study was completed by July 30, 2020. Three cancerous tissue cores and one noncancerous tissue core (1 mm in diameter) were cut lengthways from each paraffin block and installed in the new paraffin block with fine steel needles to generate tissue microarrays. This study was approved by the ethics committee of the First Affiliated Hospital of Shihezi University (No. 2019-098-01), and informed consent was obtained from each patient.

### 2.2. Immunohistochemistry

In this study, the two-step EnVision method was used for immunohistochemical experiments [23]. First, tissue sections were cut into microarrays of 4 mm, which were adsorbed on a slide. Then, fat was removed, and the tissue was rehydrated, immersed in Ethylene Diamine Tetraacetic Acid (EDTA) buffer for heat-induced antigen extraction, and immersed in 3% hydrogen peroxide to block endogenous peroxidase activity. Nonspecific antigen staining was blocked with 3% BSA. Finally, primary antibodies PLK1 (1 : 3.2 × 10^6^, ab155095, Abcam, Cambridge, UK; gastric carcinoma tissue was used as positive internal control) and AKT1 (1 : 100, ab81283, Abcam, Cambridge, UK; human cervical carcinoma tissue was used as positive internal control) were added to the slide and incubated overnight at 4°C. The next day, the tablets were redyed and sealed with hematoxylin after coloring with Diaminobenzidine (DAB) solution for 1 min. Immunohistochemical staining results were evaluated by two pathologists using a double-blind method, and the immune response score (IRS) was calculated as the percentage of positive cells multiplied by the intensity of cell staining (Table 1) [24]. According to IRS values, the results were divided into two groups, the low-expression group (<6 points) and the high-expression group (≥6 points). Section repetitions were performed when tissue chip staining was atypical [25].

### 2.3. Bioinformatics Database Validation

To improve the experiment's reliability, we used the bioinformatics database for verification. The GEPIA database (http://gepia. cancer-pku.cn/detail.php) is an online analysis website containing data from TCGA and GTEx databases for 9,736 tumor samples and 8,587 normal samples [26]. We used this database to evaluate the association between high and low expression of AKT1 and PLK1 in OSCC tissues and patient outcomes. Finally, we used the GEPIA database to verify the correlation between AKT1 and PLK1 gene expression in OSCC tissues and normal oral mucosa.

### 2.4. Statistical Analysis

The SPSS 23 software was used to analyze all experimental data in this study. The chi-square test and Fisher's exact test were used to examine the correlation between the expression levels of AKT1 and PLK1 and the clinicopathological characteristics of patients with OSCC. For survival analyses, Kaplan–Meier survival curves were constructed, and differences were tested by the log-rank test. OS was defined as the time between the date of surgery and the date of death from OSCC or the date of the last contact. The Spearman's rank correlation test was used to determine the relationships between AKT1 and PLK1 expressions. *P* value was calculated based on a two-tailed statistical analysis, and statistical significance was set at *P* < 0.05.

## 3. Results

### 3.1. Expression Rates of AKT1 and PLK1 in OSCC Tissue and Normal Oral Mucosa

**Figure 1:**
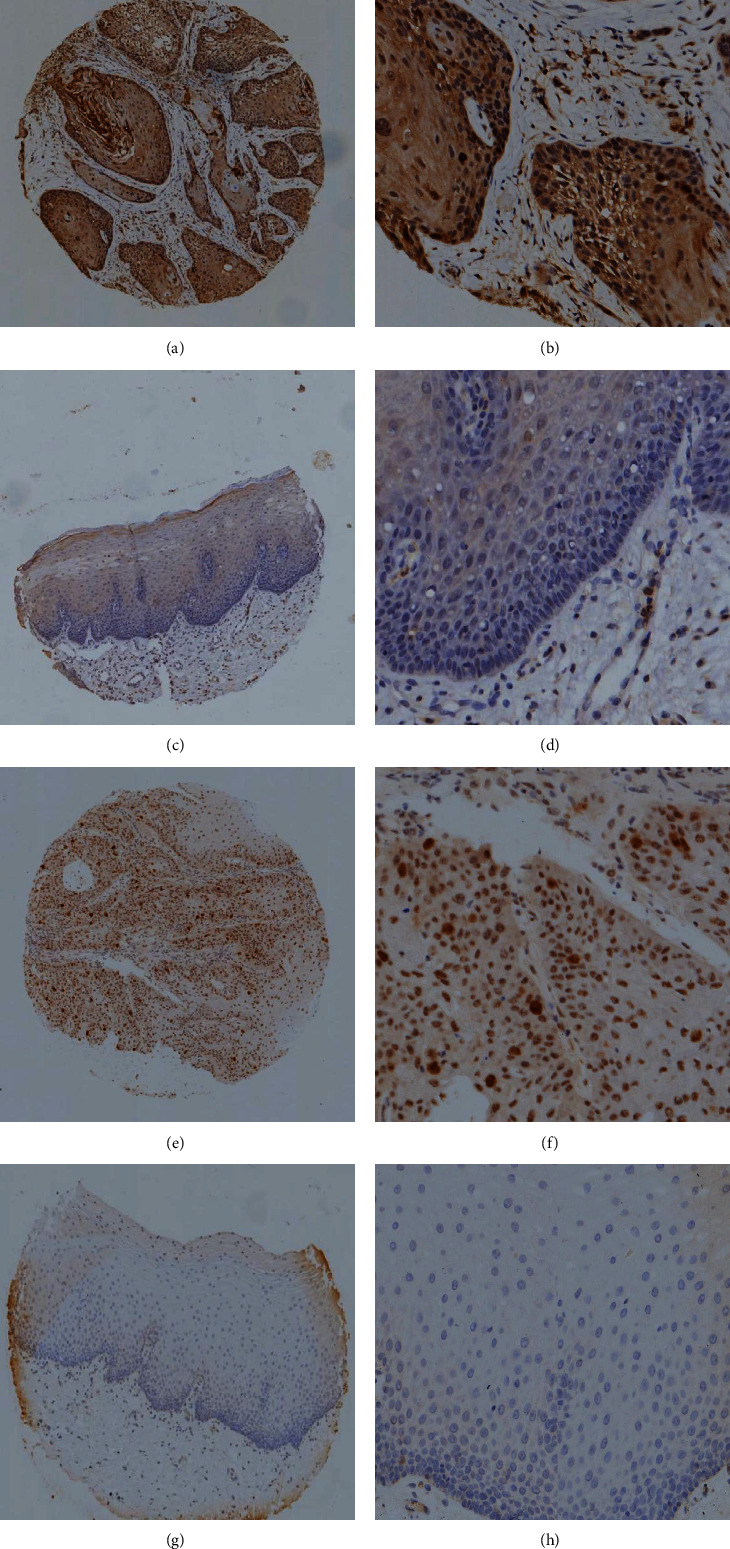
Expressions of AKT1 and PLK1 in OSCC. Expression of AKT1 in oral squamous cell carcinoma tissues: (a) original magnification ×40 and (b) original magnification ×200. Expression of AKT1 in normal oral mucosa: (c) original magnification ×40 and (d) original magnification ×200. Expression of PLK1 in oral squamous cell carcinoma tissues: (e) original magnification ×40 and (f) original magnification ×200. Expression of PLK1 in the normal oral mucosa: (g) original magnification ×40 and (h) original magnification ×200.

Immunohistochemical results showed that AKT1 was mainly distributed in the nucleus or the cytoplasm of OSCC cells (Figures 1(a) and 1(b)). In contrast, PLK1 was distributed primarily on the nucleus of OSCC cells (Figures 1(e) and 1(f)), both of which were brown or yellow-brown. There was little staining of normal oral mucosa cells (Figures 1(c) and 1(d) and 1(g) and 1(h)).

**Figure 2:**
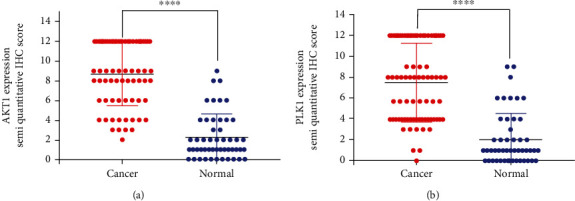
AKT1 and PLK1 expression semiquantitative IHC score. Semiquantitative IHC score of (a) AKT1 expression and (b) PLK1 expression. ^∗∗∗∗^<0.0001.

The positive expression rates of AKT1 and PLK1 in 70 OSCC tissues were 81.4% (57/70) and 58.6% (41/70), respectively. The positive expression rates of AKT1 and PLK1 in 50 normal oral mucosae were 14% (7/50) and 16% (8/50), respectively. The results showed that the expression rates of AKT1 and PLK1 in OSCC tissues were significantly higher than those in the normal oral mucosa (*P* < 0.05; Table 2 and Figures 2(a) and 2(b)).

### 3.2. Relationships between the Expressions of AKT1 and PLK1 and the Clinicopathologic Characteristics in Patients with OSCC

The expression levels of AKT1 proteins in 70 OSCC tissues had no significant correlation with patients' age, sex, tumor stage, tumor size, lymph nodes, tumor differentiation, or smoking and alcohol consumption history (*P* > 0.05; Table 3). The expressions of PLK1 in the 70 OSCC tissues showed a significant correlation with patients' tumor stage and size (*P* < 0.05). In contrast, no significant associations were observed between PLK1 expression and patient' age, sex, lymph nodes, tumor differentiation, or smoking and alcohol consumption history (*P* > 0.05; Table 3).

### 3.3. The Impact of AKT1 and PLK1 Expression on Overall Survival

**Figure 3:**
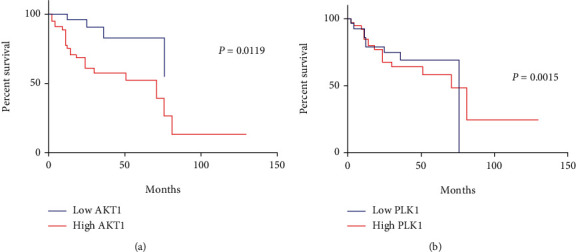
Survival analysis of AKT1 and PLK1 expression and prognosis of oral squamous cell carcinoma patients. Survival analysis of (a) AKT1 expression and (b) PLK1 expression and prognosis of oral squamous cell carcinoma patients.

To assess the prognostic impact of AKT1 and PLK1 expression in patients with OSCC, we used Kaplan–Meier survival analysis to assess the association between AKT1 and PLK1 expression and OS. The results showed that the survival time of patients with high AKT1 and PLK1 expression was statistically different from those with low AKT1 and PLK1 expression (*P* < 0.05; Figures 3(a) and 3(b)). In other words, patients with high AKT1 and PLK1 expression had a shorter postoperative survival.

### 3.4. Relationship between AKT1 and PLK1 Expression in OSCC and Normal Oral Mucosa

Immunohistochemical staining analysis of AKT1 and PLK1 showed that AKT1 and PLK1 were coexpressed in 35/70 (50%) OSCC tissues, but for 15/70 (21.4%) OSCC tissues, there was no association between AKT1 and PLK1. Correlation analysis showed that in OSCC tissues, PLK1 was positively correlated with AKT1 (*R* = 0.53; *P* < 0.0001; Table 4). In normal oral mucosa, 2/50 (4%) of normal oral mucosa had positive coexpression of AKT1 and PLK1, while 41/50 (82%) of normal oral mucosa tissues showed no association, and correlation analysis showed that PLK1 was not correlated with AKT1 (*R* = 0.19; *P* = 0.17; Table 5). Thus, there was a significant correlation between AKT1 and PLK1 expression in OSCC tissues but no correlation in the normal oral mucosa.

### 3.5. Verifying AKT1 and PLK1 Results with the Bioinformatics Database

**Figure 4:**
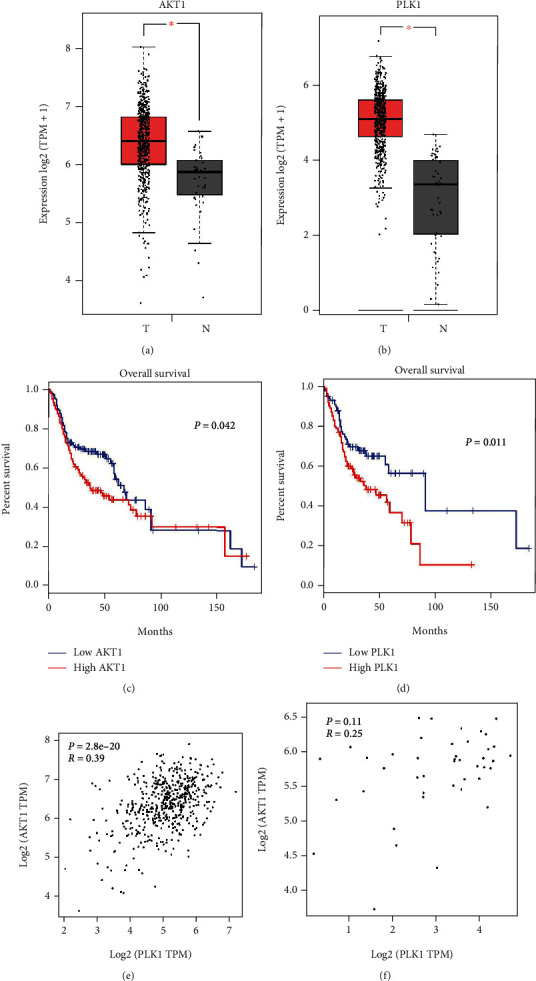
GEPIA database was used to verify the experimental results. Differences in (a) AKT1 expression and (b) PLK1 expression in oral squamous cell carcinoma tissues and normal oral mucosa. Survival analysis of (c) AKT1 expression and (d) PLK1 expression in oral squamous cell carcinoma patients. Relationship between AKT1 and PLK1 expression in (e) oral squamous cell carcinoma tissues and (f) normal oral mucosa. ^∗^*P* < 0.05.

The results of bioinformatics database verification showed that the expression of AKT1 and PLK1 in OSCC tissue was significantly higher than that in the normal oral mucosa (*P* < 0.05; Figures 4(a) and 4(b)). Similarly, the survival time of patients with high AKT1 and PLK1 expression in OSCC was significantly lower than that of patients with low AKT1 and PLK1 expression (*P* < 0.05; Figures 4(c) and 4(d)). Finally, correlation analysis of AKT1 and PLK1 showed a significant correlation between AKT1 and PLK1 expression in OSCC (*R* = 0.39; *P* = 2.8*E* − 20). On the other hand, there was no correlation between AKT1 and PLK1 expression in normal oral tissues (*R* = 025; *P* = 0.11; Figures 4(e) and 4(f)).

## 4. Discussion

Oral squamous cell carcinoma (OSCC) is the third most common cancer in developing countries and the sixth most common cancer globally [27]. Today, with the continuous development of molecular biology, it has been found that cancer was caused by genetic, metabolic, inflammatory, and epigenetic factors [28]. These lead to abnormal cell physiology and signal pathway conduction, which led to abnormal cell proliferation and differentiation, eventually developing into cancer [29].

AKT1 is a threonine/serine protein kinase. Phosphorylated AKT1 is the active form of AKT1. It is released from the cell membrane into the cytoplasm and is involved in molecular processes that promote cell growth and proliferation, such as glucose metabolism, protein synthesis, and antiapoptotic activity [30]. AKT activation depends on the PL3K pathway and is considered a key node in this pathway. AKT1 is essential for cell growth and survival [31], where high activation of AKT1 leads to excessive cell proliferation and malignant transformation [32]. Many different mechanisms mediate its activation during tumorigenesis and development. The effect of AKT1 on tumorigenesis and progression is demonstrated in a model showing that malignant tumor formation is closely related to cell infection with a retrovirus vector expressing AKT1 [33].

PLK1, a PLK subtype, is a highly conserved serine/threonine protein kinas [34]. During the DNA damage response, PLK1 enzyme activity promotes homologous recombination-mediated repair in collaboration with PARP1 and CHK1 [35]. It is clear that PLK1 is key to maintaining the normal operation of the cell cycle. PLK1 overexpression is associated with tumorigenesis. In contrast, PLK1 inhibitors act on multiple stages of cell mitosis, such as blocking centrosome maturation, spindle formation, and cytokinesis, thereby disrupting cell division and cycle progression and ultimately leading to tumor cell death [12]. Studies have shown that PLK1 is overexpressed in colorectal cancer [36], pancreatic cancer [37], gastric cancer [38], prostate cancer [39], thyroid cancer [40], bladder cancer, and other tumors [41], and the high expression of PLK1 indicates poor clinical prognosis.

After the expression of PLK1 is reduced, the proliferation of tumor cells is inhibited, and apoptosis occurs, thus preventing the occurrence and development of tumors and improving prognosis [42]. Due to this, PLK1-targeting inhibitors have attracted the attention of researchers. Some studies have shown that BI2536, Volasertib, and GSK461364 can effectively inhibit the expression of PLK1, and BI2536 has entered phase I clinical trials for colorectal cancer, liver cancer, fallopian duct cancer, and other cancers [43–45].

PI3K/Akt and PLK1 have been extensively studied in tumors, but few studies on PI3K/Akt and PLK1 in OSCC. The results of this study showed that the expression of AKT1 and PLK1 in OSCC tissues was significantly higher than that in the normal oral mucosa, which suggests the high expression of AKT1 and PLK1 may play an important role in the occurrence of OSCC. Subsequently, we analyzed the correlation between the expression of AKT1 and PLK1 in OSCC tissues and the clinicopathologic parameters of tumors. The results showed that the expression levels of AKT1 in OSCC tissues were not significantly correlated with age, sex, tumor stage, tumor size, lymph nodes, tumor differentiation, or smoking and alcohol consumption history. This may be because our sample size was small, so further expansion of our collection was warranted. However, the expressions of PLK1 in OSCC tissues were significantly correlated with tumor stage and size; but no significant associations were observed with age, sex, lymph nodes, tumor differentiation, or smoking and alcohol consumption history. These results suggested that PLK1 played an important role in the development of OSCC. Finally, we applied Kaplan–Meier analysis to determine the survival of OSCC patients. The results showed that the postoperative survival of patients in the group with high AKT1 and PLK1 expression was significantly lower than that in the group with low AKT1 and PLK1 expression, showing that AKT1 and PLK1 were closely related to the survival rate of patients with OSCC. These findings suggested that AKT1 and PLK1 can be used as prognostic markers for OSCC patients.

In addition, we also studied the relationship between AKT1 and PLK1 in OSCC tissues. Studies had shown that the upregulated expression of PLK1 can activate the PI3K/AKT signaling pathway, promote the proliferation of gastric mucosal epithelial cells, and increase the possibility of gastric cancer [46]. In addition, miR-1224-5P in osteosarcoma directly targets PLK1 through the PI3K/AKT signaling pathway to activate autophagy and cell invasion [47]. Consistent with these findings, we confirm that PLK1 and AKT1 were significantly correlated in OSCC. In other words, there was a correlation between the two, and they may promote each other's expressions.

Finally, to improve the credibility of this study, we used a bioinformatics database to verify the differential expression of AKT1 and PLK1 in OSCC tissues and normal oral mucosa, showing that the expression of AKT1 and PLK1 in OSCC was significantly higher than that in the normal oral mucosa. The survival time of patients with high AKT1 and PLK1 expression in OSCC was significantly lower than that of patients with low AKT1 and PLK1 expression. The correlation analysis of AKT1 and PLK1 in OSCC tissues and normal oral mucosa was validated. The results showed that AKT1 and PLK1 were significantly correlated in OSCC tissues but not in the normal oral mucosa. The results of the bioinformatics database validation were consistent with the experimental results, suggesting that AKT1 and PLK1 have potential research value in OSCC.

## 5. Conclusion

In conclusion, our results suggest that the expressions of AKT1 and PLK1 are closely related to the occurrence, development, and prognosis of OSCC. In addition, we found a significant correlation between the expressions of these two molecules in OSCC, which may be involved in the transformation of normal oral mucosa to OSCC. However, the details of the regulation mechanism of AKT1 and PLK1 need to be further verified by a large quantum of clinical data and long-term follow-up information combined with relevant molecular biology and cytology experiments.

## Figures and Tables

**Table 1:** Immunohistochemical score table.

Positive cells (%)		Intensity		IRS	
Percentage	Score	Color	Score	Rank	Total score
<5%	0	No color	0	-	0-1
6~25%	1	Yellow	1	+	2-4
26~50%	2	Tan	2	++	5-8
51~75%	3	Brown	3	+++	9-12
76~100%	4				

**Table 2:** Positive expression rates of AKT1 and PLK1 proteins in OSCC tissue and normal oral mucosa samples.

Protein and pathology type	Number	Positive (*M* ± SD)	Negative (*M* ± SD)	*P*
*AKT1*				
OSCC	70	57 (9.86 ± 0.30)	13 (3.54 ± 0.18)	<0.0001
Normal oral tissue	50	7 (7.00 ± 0.48)	43 (1.46 ± 0.20)	
*PLK1*				
OSCC	70	41 (10.34 ± 0.29)	29 (3.48 ± 0.19)	<0.0001
Normal oral tissue	50	8 (7.00 ± 0.50)	42 (1.61 ± 0.27)	

Annotation: *M* ± SD: mean ± standard deviation.

**Table 3:** Relationships between the expressions of AKT1 and PLK1 and the clinicopathologic characteristics in patients with OSCC.

Parameters	Number	AKT1 expression				PLK1 expression			
		Low (*M* ± SD)	High (*M* ± SD)	*χ* ^2^	*P*	Low (*M* ± SD)	High (*M* ± SD)	*χ* ^2^	*P*
*Age*									
≤60	21	6 (3.66 ± 0.21)	15 (10.07 ± 0.59)	0.94	0.33	10 (3.20 ± 0.46)	11 (10.64 ± 0.57)	0.47	0.49
>60	49	7 (3.42 ± 0.29)	42 (9.78 ± 0.35)			19 (3.63 ± 0.17)	30 (10.23 ± 0.35)		
*Gender*									
Male	37	6 (3.66 ± 0.21)	31 (9.67 ± 0.44)	0.29	0.59	15 (3.60 ± 0.27)	22 (10.64 ± 0.39)	0.03	0.87
Female	33	7 (3.42 ± 0.29)	26 (10.08 ± 0.41)			14 (3.35 ± 0.28)	19 (10.00 ± 0.45)		
*Stage*									
I+II	34	9 (3.44 ± 0.24)	25 (9.44 ± 0.49)	2.73	0.10	19 (3.42 ± 0.25)	15 (10.47 ± 0.50)	5.68	0.02
III+IV	36	4 (3.75 ± 0.25)	32 (10.19 ± 0.37)			10 (3.60 ± 0.30)	26 (10.27 ± 0.37)		
*T status*									
≤4 cm	38	9 (3.44 ± 0.24)	29 (9.58 ± 0.46)	1.44	0.23	21 (3.47 ± 0.23)	17 (10.47 ± 0.46)	6.56	0.01
>4 cm	32	4 (3.75 ± 0.25)	28 (10.14 ± 0.38)			8 (3.50 ± 0.37)	24 (10.25 ± 0.40)		
*N status*									
N0	60	11 (3.54 ± 0.20)	49 (9.75 ± 0.33)	0.16	0.69	26 (3.42 ± 0.21)	34 (10.09 ± 0.33)	0.63	0.43
N1-3	10	2 (3.50 ± 0.50)	8 (10.50 ± 0.56)			3 (3.66 ± 0.33)	7 (11.57 ± 0.42)		
*Tumor differentiation*									
Well	24	3 (3.00 ± 0.57)	21 (10.25 ± 0.37)	0.89	0.34	11 (3.44 ± 0.27)	13 (10.68 ± 0.34)	0.29	0.58
Moderate+poorly	44	10 (3.70 ± 0.15)	36 (9.19 ± 0.49)			18 (3.54 ± 0.28)	28 (9.61 ± 0.54)		
*Smoking history*									
Yes	21	3 (3.66 ± 0.33)	18 (9.66 ± 0.55)	0.36	0.55	8 (3.87 ± 0.12)	13 (11.15 ± 0.45)	0.14	0.71
No	49	10 (3.40 ± 0.22)	39 (9.94 ± 0.36)			21 (3.33 ± 0.26)	28 (9.96 ± 0.36)		
*The history of drinking*									
Yes	9	2 (3.50 ± 0.50)	7 (8.62 ± 0.88)	0.03	0.86	4 (3.75 ± 0.25)	5 (11.2 ± 0.80)	0.64	0.80
No	61	12 (3.50 ± 0.19)	49 (10.06 ± 0.31)			25 (3.40 ± 0.22)	36 (10.22 ± 0.32)		

Annotation: *M* ± SD: mean ± standard deviation.

**Table 4:** The relationships between AKT1 and PLK1 protein expression in OSCC tissue.

	PLK1 expression			
	-	+	*R*	*P*
*AKT1 expression*				
-	15	6	0.53	<0.0001
+	14	35		

**Table 5:** The relationships between AKT1 and PLK1 protein expression in normal oral mucosa.

	PLK1 expression			
	-	+	*R*	*P*
*AKT1 expression*				
-	41	6	0.19	0.17
+	1	2		

Annotation: *R* > 0.8: highly correlated; 0.5 < *R* < 0.8: moderate correlation; 0.3 < *R* < 0.5: low correlation; *R* < 0.3: irrelevant.

## Data Availability

The datasets used and/or analyzed during the current study are available from the corresponding author on reasonable request.

## Abbreviations

EDTA: Ethylene Diamine Tetraacetic Acid
IRS: Immune response score
OS: Overall survival
OSCC: Oral squamous cell carcinoma.
